# Clinical evidence continuous medical education: a randomised educational trial of an open access e-learning program for transferring evidence-based information – ICEKUBE (Italian Clinical Evidence Knowledge Utilization Behaviour Evaluation) – study protocol

**DOI:** 10.1186/1748-5908-3-37

**Published:** 2008-07-17

**Authors:** Lorenzo Moja, Ivan Moschetti, Michela Cinquini, Valeria Sala, Anna Compagnoni, Piergiorgio Duca, Christian Deligant, Roberto Manfrini, Luca Clivio, Roberto Satolli, Antonio Addis, Jeremy M Grimshaw, Pietro Dri, Alessandro Liberati

**Affiliations:** 1 Italian Cochrane Centre, Mario Negri Institute for Pharmacological Research, Milano, Italy; 2 Dipartimento di oncologia, Mario Negri Institute for Pharmacological Research, Milano, Italy; 3 Sendo Foundation, Milano, Italy; 4 Università degli Studi, Milano, Italy; 5 Zadig Scientific Publisher, Milano, Italy; 6 Mario Negri Institute for Pharmacological Research, Milano, Italy; 7 Italian Drug Agency (AIFA), Roma, Italy; 8 Ottawa Health Research Institute, Clinical Epidemiology Program, Centre for Best Practice, Institute of Population Health, University of Ottawa, Ottawa, Canada

## Abstract

**Background:**

In an effort to ensure that all physicians have access to valid and reliable evidence on drug effectiveness, the Italian Drug Agency sponsored a free-access e-learning system, based on *Clinical Evidence*, called ECCE. Doctors have access to an electronic version and related clinical vignettes. Correct answers to the interactive vignettes provide Continuing Medical Education credits. The aims of this trial are to establish whether the e-learning program (ECCE) increases physicians' basic knowledge about common clinical scenarios, and whether ECCE is superior to the passive diffusion of information through the printed version of *Clinical Evidence*.

**Design:**

All Italian doctors naïve to ECCE will be randomised to three groups. Group one will have access to ECCE for *Clinical Evidence *chapters and vignettes lot A and will provide control data for *Clinical Evidence *chapters and vignettes lot B; group two vice versa; group three will receive the concise printed version of *Clinical Evidence*. There are in fact two designs: a before and after pragmatic trial utilising a two by two incomplete block design (group one versus group two) and a classical design (group one and two versus group three). The primary outcome will be the retention of *Clinical Evidence *contents assessed from the scores for clinical vignettes selected from ECCE at least six months after the intervention. To avoid test-retest effects, we will randomly select vignettes out of lot A and lot B, avoiding repetitions. In order to preserve the comparability of lots, we will select vignettes with similar, optimal psychometric characteristics.

**Trial registration:**

ISRCTN27453314

## Background

### Continuing Medical Education for health professionals

In the last decade many countries have legislated within their health systems the revalidation and recertification of medical practitioners [1]. Two principles underlie Continuing Medical Education (CME): professional development is a process of lifelong learning in practice, and professionals must be able to demonstrate they are clinically competent in certain roles. Many countries are facing the challenge of building a formal, sustainable assessment of skills through education programs, accredited providers, acquisition of credit points, and voluntary or mandatory regulation[1]. Although it has been claimed that CME should also include managerial, psychosocial and communication skills to reflect the multidisciplinary context of patient care[2], usually professional development is mainly based principally on advanced clinical subjects. Traditional knowledge transfer formats employ live interactive classes supported by paper-based text and graphic materials; online learning is increasing popular, supported by screen-based text and graphics with different levels of interactivity.

### CME in Italy

A compulsory system of CME was introduced in Italy for all health professionals in 1998. During the five-year cycle from 2002 to 2006, health professionals have been required to earn a total of 150 credits [3]. A national education committee defines the educational standards, accredits educational events, and sets the number of credits earned. A credit involves one hours' learning, *i.e*., one hour of education is worth one credit, but there is a weighting towards activities that present evidence-based (EB) information and are free of commercial interests. The traditional form of acquiring CME credits is to attend lectures and conferences; it is more rare for doctors to be exposed to small group interactive events. Between 2004 and 2005, the committee approved and evaluated the provision of distant learning programmes: 136,208 health professionals (46,952 doctors, 34%) earned 737,603 credits, a result welcomed as a large success [4].

### Dissemination of independent information in Italy

**Figure 1:**
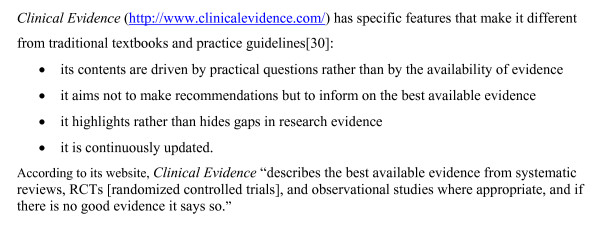
Clinical Evidence.

In an effort to ensure that all physicians have open access to valid and reliable evidence on drug effectiveness and safety, the Italian Drug Agency (AIFA) launched a program to disseminate independent, unbiased information. The agency did this by translating into Italian *Clinical Evidence*, a compendium of the best available evidence on treating a wide range of common conditions (Figure 1), and freely distributing it. By 2006, the fourth Italian edition (based on *Clinical Evidence*, Vol. 14) had been published. The online version is freely available to all practicing 248,000 doctors, and 47,000 general practitioners (GPs) receive also a free copy of the concise version of the book.

Interest in EB health care is growing in Italy, although its impact upon clinical practice is still not clear. In 1999, the first pilot free distribution of 50,000 copies of *Clinical Evidence *was assessed through a survey exploring doctors' judgement of its validity, relevance, and usability. The findings indicated that the compendium had been well-received, and confirmed doctors' preference for problem-driven information and the key role of strong endorsement from health authorities for its implementation [5]. However, another survey funded by the Smith Kline Foundation showed that Italian GPs were satisfied with the information provided by pharmaceutical sales representatives, who they considered complete and sufficiently reliable [6].

**Figure 2:**
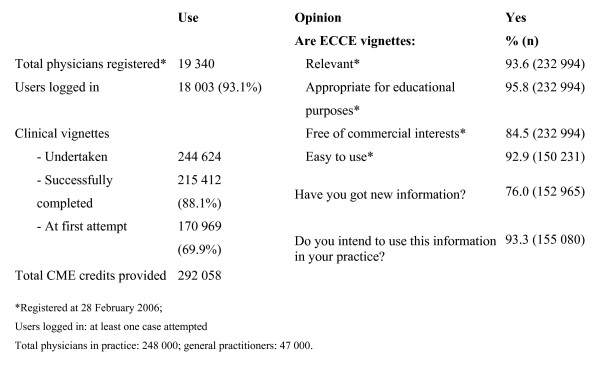
**Use and average respondents' opinions of ECCE by registered doctors**[7].

In order to make certain it was worth the expense of disseminating *Clinical Evidence *and to speed up the diffusion of EBM, AIFA sponsored a free-access e-learning system, based on *Clinical Evidence*, called ECCE (the Italian acronym for Continuing Education Clinical Evidence). ECCE became accessible to all physicians in March 2005 after a pilot period. The results of the first year of use (until February 2006) are reported in detail elsewhere and summarised in Figure 2[7].

### The potential role of e-learning from transferring information to modifying clinical practice

Several conceptual models have been developed for assessing knowledge and competence. A popular one proposed by Miller identifies four stages of development: 'knows, knows how, shows how, and does' that are the essential facets of clinical competence [8]. An individual progresses through cognitive and behavioural steps, from acquiring knowledge to performing a task in practice. Miller's theory assumes that competence predicts performance. Other proposed models consider other influences on a doctor's performance [9], including system-related factors (government incentives, guidelines) and individual-related ones (patient's expectation, relationship with peers) with increasing levels of complexity.

**Figure 3:**
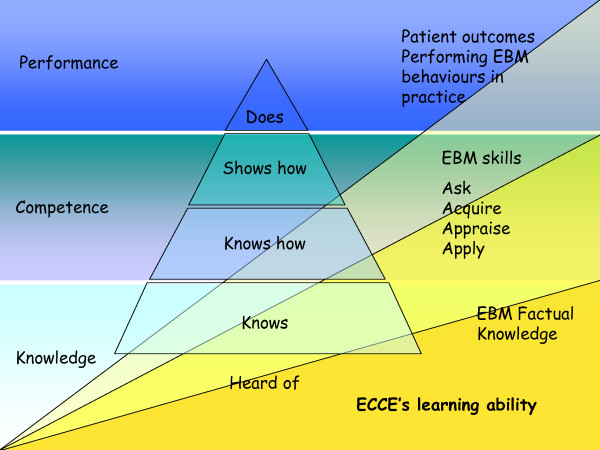
**Miller's model modified to consider different steps of knowledge, competence and performance of EBM**. In this model, the learning ability of ECCE decreases from knowledge to performance (in yellow).

For the purposes of this trial we will adopt Miller's simpler model. The stages of development have been slightly changed to better reflect the different components and skills targeted by educational interventions focusing on EBM: 1) factual knowledge or basic learning: knowing the benefits and risks of different interventions (*e.g*., in patients with unstable angina, aspirin is beneficial); 2) deep learning or competence: posing structured clinical questions considering patients, treatment, comparison, and outcomes and understanding quantitative aspects (relative or absolute risk reduction, number needed to treat); 3) point-of-care ability or performance: the incorporation of EB information into practice, with the adoption of proven treatments and interventions that can potentially improve patients' health. The modified EBM Miller's model is shown in Figure 3.

ECCE is thought to directly affect superficial learning (ability to reproduce facts) and, with decreasing impact, deep learning (ability to apply concepts and skills in the workplace) [10]. ECCE's impact on clinical behaviour and patients' outcomes is less straightforward: we prefer to adopt a conservative framework considering phases that necessarily follow one another [11]. Therefore, in this study we want first and foremost to show an improvement in physicians' knowledge, which may or may not affect competence and, finally, on behaviours and patients' outcomes.

### Aim of the study

This trial will test the effectiveness of ECCE e-learning program for transferring EB information to medical doctors after a median of three months of ECCE usage, and retention of the information after a median period of six months. The main hypotheses to be tested are: 1) Does an e-learning CME program based on *Clinical Evidence *and clinical vignettes (ECCE) increase physicians' basic knowledge about epidemiology, therapy, prognosis, and risk factors in a clinical scenario? 2) Do physicians retain the knowledge from the ECCE for more than six months? 3) Is this educational intervention superior to the passive diffusion of information through the printed version of *Clinical Evidence*?

The secondary hypotheses are: Does ECCE modify physicians' attitudes to EB behaviours in a simulated realistic clinical scenario?

## Methods

### ECCE: An E-learning CME Program

ECCE is an e-learning CME tool that uses interactive clinical vignettes based on chapters in *Clinical Evidence *and a predefined sequence of questions. ECCE has four components: 1) the *Clinical Evidence *chapter (*e.g*., headache, chronic tension-type); 2) a clinical vignette from the *Clinical Evidence *chapter that presents a plausible medical scenario (*e.g*., Margaret says to her family doctor: "This time I didn't come for me, but to talk about Rachel, my 25-year-old daughter....); 3) questions addressing the recall of *Clinical Evidence *facts or their application to the medical scenario, from which the doctor is to select the correct answer; 4) the potential answers (*e.g*., a list of potential efficacy descriptors for a therapeutic regimen relevant to the theme); and 5) instructions on what to do (*e.g*., "more than one answer may be correct").

**Figure 4:**
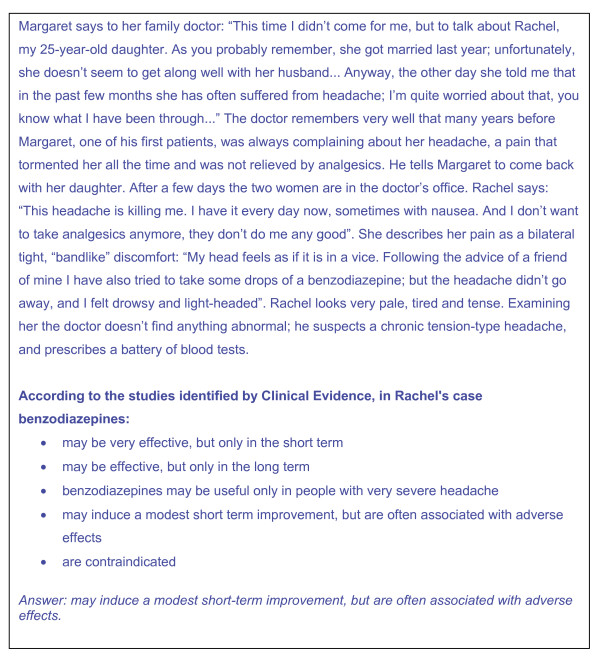
**Example of an ECCE vignette.** We present the first step of the headache (chronic tension-type) vignette and related questions. The vignette was developed from Headache (chronic tension-type), Clinical Evidence.

Figure 4 gives an example of a vignette from ECCE. The response option includes one or more correct answers for each question, and other reasonably plausible but incorrect response as distractors.

Vignettes are intended to replicate real-life circumstances as seen by an ordinary GP in everyday practice. Whilst GPs are the primary target of ECCE, many vignettes are also relevant to specialists. Each vignette has a narrative with events and clinical details presented in chronological order: the history evolves with new information from diagnostic tests or additional information reported by the patient. All vignettes use news media techniques, sometimes with fictional or interactive elements (*e.g*., mystery fiction, the possibility to order tests and obtain results in real time, test appropriateness and cost). Users solve the single steps though a question and answer decision system. They gain credits upon completing all steps where they reach a score of 80% or more of the total. Vignettes provide one or two credits depending on the number of questions. Users are required to finish the vignette started regardless of the score reached. If a user fails, the vignette is then not used again for 24 hours. In 2005, we posted 120 vignettes, and in 2006, 52 new ones.

Both clinical vignettes and related questions will be carefully planned against *Clinical Evidence *chapters by ECCE authors who are all specialised medical practitioners. Standardised guidelines will be developed by the ECCE editors to help authors. Author guidelines will consider system rules (*i.e*., each question has five multiple-choice answers), writing style (avoid misleading constructs such as double negatives), medical style (avoid excessive technical jargon, such as *rigor nucalis*) and provide an example framework. Each vignette will be revised by two editors to ensure it is appropriate for the *Clinical Evidence *chapter tested. All vignettes will be copy-edited to high editorial standards. Although this would be a highly standardised process, a skilled clinical vignette writer is essential for valid assessment of doctors' abilities and competencies [12].

ECCE has all the standard advantages of e-learning. Users select what and when they want to learn, and at what pace. The system is easy to use and works with basic computer requirements (*e.g*., low speed connection). The contents of *Clinical Evidence *can be read on-screen or printed and interactively managed along the steps of each vignette. The system tracks learning content and the learner's progress.

### Study design

This is a before and after pragmatic randomised controlled trial utilising a two-by-two incomplete block design. When evaluating educational interventions aimed at improving clinical practice, a number of non-specific effects may influence estimates of the effect of an intervention, grouped together under the term Hawthorne effect [13-17]. These include positive attention effects, caused by participants knowing that they are the subject of a study, but also negative and demotivating effects, caused by being allocated to a control rather than an intervention group. If these non-specific effects are imbalanced across study groups in a quality improvement trial, the estimates may be biased. Randomised controlled trials using balanced incomplete block designs should balance such non-specific effects [13,15,17-20].

Because the Hawthorne effect may influence the outcome, this trial will adopt a two-by-two balanced incomplete block design in which subjects will be randomised into two groups. Group one will have access to ECCE for *Clinical Evidence *chapters and vignettes lot A and will provide control data for *Clinical Evidence *chapters and vignettes lot B. Group two will have access to ECCE for lot B and will provide control data for lot A. The design will be balanced because it ensures that all participants receive the same intensity of educational intervention and data collection, which should therefore balance any non-specific effects. The design will be incomplete because not all participants receive the complete education for all chapters and vignettes [14,17]. To check for a possible Hawthorne effect we will add a third control arm (classical design), with only a minimal intervention consisting of one of the elements of the complete intervention, namely the concise printed version of *Clinical Evidence *and access to the on-line full-text version. This group will not have access to the ECCE platform nor the clinical vignettes related to *Clinical Evidence*. The incomplete block design will answer the question "Does ECCE increase physicians' basic knowledge about EB information," while the classical design will answer the question "Is ECCE superior to the printed version of *Clinical Evidence." *Table 1 summarises the block and the classical designs.

**Table 1:** Designs, hypotheses per trial, and possibility of ensuring equal Hawthorne effect across the arms.

Trial comparison	Design	Hypothesis	Hawthorne
Arm I vs. II Complete intervention on lot A. Using lot B as control.	Block	If the test scores related to lot A vignettes increase in accordance with *Clinical Evidence *chapters A and there is no change in test scores of B chapters, the intervention has a genuine effect.	No
Arm II vs. I Complete intervention on lot B. Using lot A as control.	Block	If the test scores related to lot B vignettes increase in accordance with *Clinical Evidence *chapters B and there is no change in test scores of A chapters, the intervention has a genuine effect.	No
Arm I vs. III Complete intervention for lot A (ECCE). Minimal intervention on A-B chapters (*Clinical Evidence*) (control).	Classical	If the test scores related to A vignettes in the intervention arm increase in accordance with the *Clinical Evidence *chapters A and there is no change in the control arm the intervention (ECCE) has a favourable effect, but without controlling for the Hawthorne effect.	Yes
Arm II vs. III Complete intervention for lot B (ECCE). Minimal intervention on A-B chapters (*Clinical Evidence*) (control).	Classical	If the test scores related to lot B vignettes in the intervention arm increase in accordance with the *Clinical Evidence *chapters B and there is no change in the control arm the intervention (ECCE) has a favourable effect, but without controlling for the Hawthorne effect.	Yes

### Equivalence of clinical vignettes

We will develop two comparable lots of clinical vignettes focusing on different medical topics. In order to preserve comparability we will select vignettes with similar, optimal psychometric characteristics [21-23]. They will be ranked for the following psychometric properties: validity of users' judgement, content validity, internal consistency, and responsiveness.

To avoid contamination, we will make sure that the vignettes in lots A and B are different so that learning about the management of lot A conditions does not influence management of lot B conditions. For example, there may be contamination if lot A focuses on ischemic heart disease (and highlights the need to optimize diabetes control in diabetics) and lot B focuses on diabetes (and highlights the need to optimize control of ischemic heart disease risk factors).

**Figure 5:**
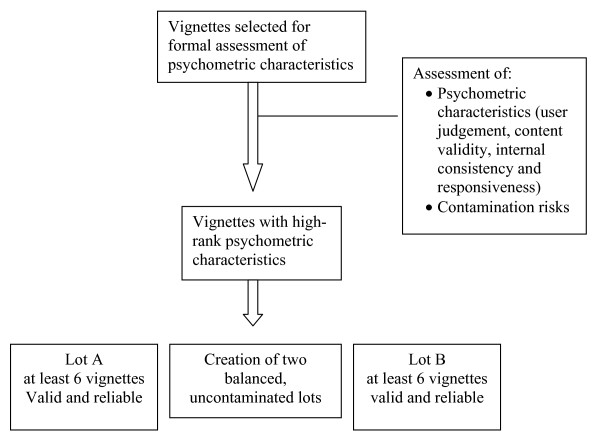
The flow of vignette selection through the different phases.

The authors will analyse the cumulative profile of the psychometric characteristics of each vignette and will select valid, reliable, and distinct vignettes. These will then be balanced to the intervention arms to have two comparable lots, avoiding contamination. The flow of vignette selection through the different phases is presented in Figure 5. We cannot estimate how many vignettes will reach the threshold for validity and reliability to be considered appropriate. We will stop selection only when we have at least six appropriate vignettes for the two ECCE arms in the incomplete block design.

### Study participants

This study will involve Italian doctors naïve to ECCE who voluntarily agree to participate. Doctors can learn about this research program through advertisements in medical journals and websites. New users who enter the ECCE website will be automatically invited to participate. There will be no exclusion criteria.

### Allocation to experimental arms

**Figure 6:**
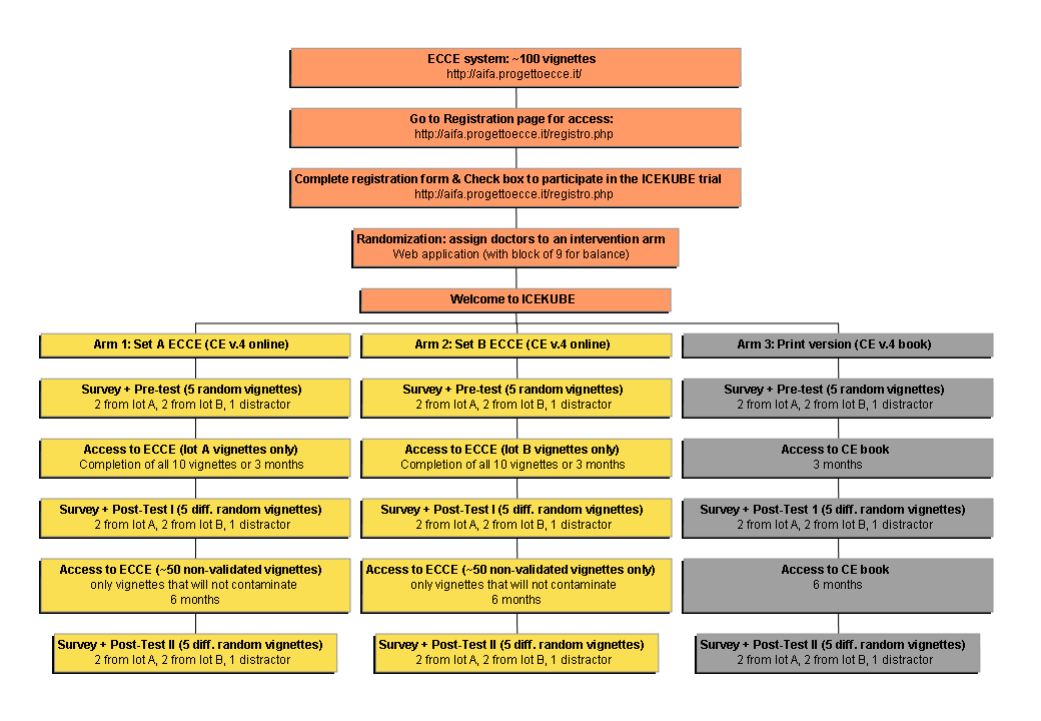
The flow of participants through the different trial steps.

The Italian Cochrane Centre will produce the computer algorithm for the allocation sequence and will implement it on ECCE. Once doctors agree to participate, ECCE will assign doctors to one of the three arms using a balanced randomisation scheme. Researchers will be unable to manipulate the randomisation sequence or interfere with the ECCE registration process. In Figure 6, we present a flow chart outlining the progress of participants through the study. This protocol follows the recommendations of the Consort Statement [Additional file 1] [24].

### Intervention

Doctors randomised to ECCE will have access to their clinical vignettes for three months after enrolment, or until they finish all vignettes. All participants will have a logbook to indicate how many times they accessed the platform and how much time they spent solving clinical vignettes. Doctors randomised to receive a printed copy of *Clinical Evidence *may use it as much as they want over the whole trial period and may have access to the on-line version of *Clinical Evidence*. The ECCE platform will always be inaccessible to them during the intervention period. After the intervention period, a sample of contamination-controlled clinical vignettes will be accessible to arm 1 and 2 participants until the end of the trial.

### Outcomes measures and measurements

The primary outcome will be basic knowledge of the *Clinical Evidence *contents assessed through the scores from the clinical vignettes. The test will consist of fixed and multiple-choice questions from the selected valid, reliable vignettes and will be administered before (pre-test), immediately after (approximately 16 weeks after enrolment, post-test one), and six months after the intervention (approximately 36 weeks after enrolment, post-test two) (see Figure 6).

To reduce the risk of a test-training effect (practice effects or memory of the first administration will influence post-test performance) at each test section ('pre-test', 'post-test one' and 'post-test two'), we will randomly select two vignettes from lot A and two from lot B to avoid repetition. Therefor, each participant will have only a limited probability (approximately zero) of test/re-testing the same vignette from one assessment to another. The questionnaire will investigate the same *Clinical Evidence *knowledge for intervention and control chapters. This random sampling of outcomes with high psychometric standards will eliminate any interactions between the causal relationship and the classes of outcomes studied or not studied within the same group of vignettes [25]. To prevent participants from guessing which vignettes will be evaluated, we will add a number of "distracter" vignettes to lots A and B. All groups will also rate their satisfaction with the information source and its perceived value for their medical education and clinical practice.

### Study procedures and data collection

The three arms will complete a pre-activity demographic survey, the knowledge tests, and surveys of participation at other educational events. There is growing recognition of EBM in Italy and independent educational initiatives to improve general practice are becoming more common. During the trial, this additional education could be an important threat to internal validity. We will record these concurrent educational experiences in our sample and assess their effects in exploratory post hoc analyses.

Data will be collected from all participants using online instruments. The ECCE platform will manage and track all data collection, following up by e-mail the participants after 16 and 36 weeks. Although researchers are not blinded for the trial group allocation, they will not be able to interfere in collection.

The demographic survey will record sex, age, and years of practice; practice characteristics; preferred CME format; computer skills; ease in using the Internet; familiarity with EB information; frequency of attending residential and distant CME activities.

### Sample size and calculation of power

Based on a preliminary examination of test scores, we established that the smallest useful difference for this intervention was a 20% absolute improvement. Based on a preliminary test of 300 doctors, we found that a 28% absolute improvement was due to the intervention. We standardised the absolute improvement across different type of vignettes (five or ten questions). Therefore we calculated our sample size to detect a 0.7 standardized difference in the primary outcome, set the α error rate at 0.05 (two-sided), and the β error at 0.10 (90% power). This yielded a sample size of 45 practitioners per study arm. If the accrual period is six months, and the maximum follow-up period is six months with a loss during follow-up of 20% at the end of the study, the total number of practitioners to be randomised has been adjusted upwards to 162 (54 per intervention group).

### Statistical analyses

Knowledge test data will be analysed using repeated-measure analyses of variance (ANOVA), reporting the partial omega squared (ù2) effect size with corresponding 95% confidence interval. Scores for the    knowledge test scale will be subjected totwo-by-three [TO TWO-BY-THREE] repeated-measures ANOVA having: 1) one between-subjects factor (ECCE lot A and ECCE lot B) and one within-subject factor (pre-test, post-test one and post-test two) for the incomplete block design trial; 2) one between-subjects factor (ECCE set and control) and one within-subject factor (pre-test, post-test one and post-test two) for the classical trial. Tests of Sidak-adjusted simple main effects will be used for post-hoc mean comparisons as needed. Orthogonal planned contrasts will be formulated for the knowledge test data to verify knowledge retention. We do not expect the data sets to follow normal distributions and assumptions completely. If this is the case, we will do nonparametric and robust analyses to explore differences in medians or distributional shapes. If nonparametric analysis yields similar interpretations and conclusions, we will focus on the parametric results.

Additional analyses will include examination of the relationship between knowledge test outcomes and potential moderator variables including sex, age, and years of practice, speciality (*e.g*., general internal medicine versus general surgery); preferred CME format; computer skills; ease using the internet; familiarity with and readiness to use EB information; study drop-out; satisfaction with learning experience, and frequency of attending residential and distant CME activities. For these analyses, we will use parametric methods (regression, repeated-measures ANOVA, analyses of covariance (ANCOVA)) and nonparametric methods (Wilcoxon-Mann-Whitney test, χ^2 ^test of independence and γ measure of association) as needed, based on the data. Holm's modified Bonferroni corrections will be applied to check experimental error (*e.g*., in exploratory analyses). Analyses will be done based on an intention-to-treat basis, whether or not doctors logged and took credits. We will restrict the explanatory analysis to those doctors who took credits. All the analyses will consider *p *= 0.05 as significant (two-sided).

SAS version 9.0 statistical software (SAS Institute, Inc, Cary NC) will be used.

### Ethical approval

The study has been approved by the Research Ethics Board Azienda Sanitaria Locale "Città di Milano", Milano (file number 43-06 SO) and the participating institutions (Italian Drug Agency, the Mario Negri Institute for Pharmacological Research and the Italian Cochrane Centre). This study is funded by a grant from the Italian Drug Agency, a government agency that aims to provide credible, impartial advice, and EB information about the effectiveness of drugs. The Italian Drug Agency approved the design and the methods but had no role in its conduct, analysis, interpretation, or reporting, and will not have access to the data. This trial is completely independent from the BMJ Publishing Group, which publishes the original version of *Clinical Evidence*.

## Discussion

This protocol of an incomplete block design randomised control trial aims to determine the effect of a large-scale online educational intervention using vignettes based on *Clinical Evidence*. The educational intervention, ECCE, comprises of:*Clinical Evidence*, the source of the content; clinical vignettes as the educational model; the e-learning tool as information and test vehicle. Our implementation of these elements may reduce the generalizability of this intervention. In addition, differences in the CME system regulation, the availability CME programmes, and access to EB health care information are likely to increase the differences in many industrialized countries. However, other elements favour the transferability of ECCE: the worldwide diffusion and appreciation of *Clinical Evidence*, including the standardization of its contents and of the clinical vignettes, its basic computer requirements, and the low cost for each credit provided.

A recent randomised controlled trial tested an internet-based CME program compared with a live course [26]. A cholesterol guideline package was delivered in these two ways and the trial assessed the long-term outcomes. Knowledge immediately before and 12 weeks after the intervention was assessed. The percentage of high-risk patients who had appropriate lipid panel screening and pharmacotherapy according to guidelines was documented with chart audits. Both interventions produced similar and significant 12-week knowledge gains. Chart audits indicated high baseline screening rates in all patient groups (93%) with no significant post-intervention change. However, the internet-based intervention was associated with a significant increase in the percentage of high-risk patients treated with drugs according to cholesterol guidelines. Our trial does not consider behavioural outcomes because this would be rather complicated due to the large number and types of diseases considered in each *Clinical Evidence *chapter. It is clearly easier to measure an educational intervention designed to change a single behaviour than one targeting general management of conditions or patients. In particular, without evidence of prior efficacy or knowledge, it may be injudicious and precipitous to consider behavioural outcomes. Indeed, our trial's positive results may reflect physicians' competence more than appropriate clinical practice. The teaching properties of case histories are known [27-29], and in a recent study vignette scores appeared to be highly correlated to physicians' practice in outpatient settings and were a valid overall measure of the care provided [30]. Furthermore, one of the greatest barriers to reading *Clinical Evidence *was boredom. If our intervention is effective, the written case simulation will turn the passive reading into a more interactive experience in which doctors search for the right information for specific situations, increasing their knowledge.

## Competing interests

The Italian Cochrane Centre (ICC) was the recipient of grants from the Italian Drug Agency (AIFA) for the translations of Clinical Evidence. Zadig developed and managed the ECCE platform and its contents and AIFA supported these activities. The ICC and Zadig closely collaborated in the two projects. The ICC was the recipient of a grant to design and carry out this trial independently from AIFA and the BMJ Publishing Group. Both the ICC and Zadig have scientific and cultural interests in the success of the dissemination of Clinical Evidence and ECCE and this may have influenced the conduct of this study.

## Authors' contributions

LM and IM specified null hypotheses and lead on study design, with contributions from MC, PD, AL, PD and JMG. LM, IM, MC, VS, AC, LC, CD and PD managed clinical vignettes' data and surveys. MC lead the statistical analyses, with contributions from LM, IM and PD. IM, PD, LC and CD managed the clinical vignettes' platform. PD, AL, RS and AA supervised the CME program. LM drafted the paper, with contribution from all authors.

## Supplementary Material

Additional file 1 CONSORT checklist. CONSORT checklist as provided by investigators to improve the reporting of the RCT. Click here for file
