# Supplementary material for: Clinical evidence continuous medical education: a randomised educational trial of an open access e-learning program for transferring evidence-based information – ICEKUBE (Italian Clinical Evidence Knowledge Utilization Behaviour Evaluation) – study protocol
Source: Implement Sci. 2008 Jul 17;3:37. doi: 10.1186/1748-5908-3-37 (PMC2494552; doi:10.1186/1748-5908-3-37)
Supplement: Additional file 1 — CONSORT checklist. CONSORT checklist as provided by investigators to improve the reporting of the RCT. [file 1748-5908-3-37-S1.doc]

CONSORT checklist for RCT protocols

| PAPER SECTION And topic | Item | Description | Reported on page # |
| --- | --- | --- | --- |
| *TITLE & ABSTRACT* | 1 | [How participants were allocated to interventions](http://www.consort-statement.org/Statement/examples1.htm) (e.g., "random allocation", "randomized", or "randomly assigned"). | 2 |
| *INTRODUCTION* Background | 2 | [Scientific background and explanation of rationale.](http://www.consort-statement.org/Statement/examples2.htm) | 3-5 |
| *METHODS* Participants | 3 | [Eligibility criteria for participants](http://www.consort-statement.org/Statement/examples3a.htm) and the [settings and locations where the data were collected](http://www.consort-statement.org/Statement/examples3b.htm). | 8 |
| Interventions | 4 | [Precise details of the interventions intended for each group and how and when they were actually administered.](http://www.consort-statement.org/Statement/examples4.htm) | 8 |
| Objectives | 5 | [Specific objectives and hypotheses](http://www.consort-statement.org/Statement/examples5.htm). | 5 |
| Outcomes | 6 | [Clearly defined primary and secondary outcome measures](http://www.consort-statement.org/Statement/examples6a.htm) and, when applicable, any [methods used to enhance the quality of measurements](http://www.consort-statement.org/Statement/examples6b.htm) (e.g., multiple observations, training of assessors). | 9 |
| Sample size | 7 | [How sample size was determined](http://www.consort-statement.org/Statement/examples7a.htm) and, when applicable, [explanation of any interim analyses and stopping rules](http://www.consort-statement.org/Statement/examples7b.htm). | 9-10 |
| Randomization -- Sequence generation | 8 | [Method used to generate the random allocation sequence](http://www.consort-statement.org/Statement/examples8a.htm), including [details of any restriction](http://www.consort-statement.org/Statement/examples8b.htm) (e.g., blocking, stratification). | 8 |
| Randomization -- Allocation concealment | 9 | [Method used to implement the random allocation sequence](http://www.consort-statement.org/Statement/examples9.htm) (e.g., numbered containers or central telephone), clarifying whether the sequence was concealed until interventions were assigned. | 8 |
| Randomization -- Implementation | 10 | [Who generated the allocation sequence, who enrolled participants, and who assigned participants to their groups.](http://www.consort-statement.org/Statement/examples10.htm) | 8 |
| Blinding (masking) | 11 | [Whether or not participants, those administering the interventions, and those assessing the outcomes were blinded to group assignment.](http://www.consort-statement.org/Statement/examples11a.htm) If done, [how the success of blinding was evaluated](http://www.consort-statement.org/Statement/examples11b.htm). | 9 |
| Statistical methods | 12 | [Statistical methods used to compare groups for primary outcome(s)](http://www.consort-statement.org/Statement/examples12a.htm); [Methods for additional analyses,](http://www.consort-statement.org/Statement/examples12b.htm) such as subgroup analyses and adjusted analyses. | 10 |
